# Age estimation using canine pulp volumes in adults: a CBCT image analysis

**DOI:** 10.1007/s00414-019-02147-5

**Published:** 2019-08-30

**Authors:** Shakeel Kazmi, Scheila Mânica, Gavin Revie, Simon Shepherd, Mark Hector

**Affiliations:** Dundee Dental School, University of Dundee, Park Place, Dundee, Scotland DD1 4HN UK

**Keywords:** Forensic odontology, Canine pulp volumes, Age estimation, Homogenous age distribution, Cone beam computed tomography

## Abstract

Secondary dentine deposition is responsible for the decrease in the volume of the pulp cavity with age. Therefore, the volume of the pulp cavity can be considered as a predictor for estimating age. The aims of this study were to investigate the relationship strength between canine pulp volumes and chronological age from homogenous (approximately equal numbers of individuals in each age range) age distribution and to assess the effect of sex as predictor in age estimation. This study was performed on 719 subjects of Pakistani origin. Cone beam computed tomography images of 521 left maxillary and 681 left mandibular canines were collected from 368 females and 349 males aged from 15 to 65 years. Planmeca Romexis® software was used to trace the outline of the pulp cavity and to calculate pulp volumes. Regression analysis was performed to assess the correlation between pulp volumes considering with and without sex as a predictor with chronological age. The obtained results showed that mandibular canine pulp volume and sex have the highest predictive power (*R*^2^ = 0.33). The relationship between mandibular canine pulp volume and sex with chronological age demonstrates an odd S-shaped non-linear relationship. A statistically significant difference in volumes of pulp was found (*p* = 0.000) between males and females. The conclusion was that predictions using the pulp volume of the mandibular canine and sex produced the best estimates of chronological age.

**Electronic supplementary material:**

The online version of this article (10.1007/s00414-019-02147-5) contains supplementary material, which is available to authorized users.

## Introduction

Teeth are preferred in age estimation methods because they are less influenced by nutritional, hormonal, and environmental factors than bone [1–3]. Dental age estimation methods which rely on patterns of the tooth development and eruption have historically proven useful for estimating the chronological age in deciduous and permanent dentitions [4–6]. Tooth development and eruption is a reliable indicator for estimating age up to the age of 16 years. From 16 to 24 years, only third molar development can be assessed but there is significant variability, the accuracy is controversial and third molars are not always present [7].

When the tooth eruption is complete, secondary dentine deposition commences and the size of the pulp cavity deceases with age. This correlation was first investigated by Bodecker in 1925 [8]. In 1952, Gustafson introduced an invasive method for age estimation based on six age-related changes, including secondary dentine [9]. Further, non-invasive studies used conventional dental radiographs of tooth to measure the pulp-tooth linear measurements and area ratios to estimate the age [10, 11]. The results by Kvaal et al. revealed that pulp-tooth linear measurements ratio were the best correlated with age with an *r*^2^ ranging from 0.56 to 0.76 [10]. Cameriere et al. introduced a method for estimating age based on pulp-tooth area ratio and obtained a value of (*r*^2^) 0.85 [11]. These two methodologies are reproducible and non-destructive in nature therefore applied to different populations [12–16]. Permanent canines have been used in many studies because these teeth have larger pulp dimensions, subject to less wear from diet and demonstrate high level of survival compared with other teeth in dentition [3, 17–19].

Several 2D radiographic studies have estimated the age based on the pulp-tooth linear measurements and pulp-tooth area ratios [12–16]. Unfortunately, superimposition of structures upon one another, failure to assess the pulp changes, and recognition of overall shape of the tooth are the main limitations of these radiographs. In other words, these radiographs provide the two-dimensional views of the three-dimensional object [20]. To overcome these limitations, 3D technology is introduced into the dentistry which enabled researchers to comprehensively evaluate the decrease in the pulp cavity caused by the formation of the secondary dentine. These images are typically composed of microcomputed tomography (μCT) and cone beam computed tomography (CBCT) images providing opportunity to measure the volumes of the pulp and tooth non-invasively and so investigate relationship, correlation with age [21–25].

Vandevoort et al. were the first to report the pulp-tooth volume ratio association with age using μCT scanning. The results revealed a moderate correlation with a linear relationship [21]. Later on, studies reported the higher coefficient of determination with linear and non-linear relationships between pulp-tooth volume ratios and age [22, 23]. Yang et al. used CBCT scans of single-rooted teeth and reported a moderate correlation with linear relationship between pulp-tooth volume ratio and age [24]. Additionally, further studies reported a different strength of correlations between pulp-tooth ratio and age with linear and non-linear relationships [1, 19, 24].

The majority of the studies based on the assessment of pulp-tooth area and volume ratios have reported that there is no difference between males and female in regression models [3, 12, 17, 19, 25, 26]. However, few studies reported that regression models are more accurate in females as compared with men [20, 22, 27]. All these studies compared the difference between sexes without considering sex as a predictor in regression models.

Age estimation research is highly affected by the number of individuals in each group and selected age range in sample size [18, 28, 29]. Most of the studies lack the uniform distribution of sample size; therefore, a large data sample size characterized by homogenous age distribution (i.e., approximately equal numbers of individuals in each age range) was used in this study to estimate the age and correlation from pulp volumes of canines. In addition, sex was considered as predictor to evaluate its effect on age estimation.

More recently, a new method based on calculating pulp volume alone was introduced into 3D studies for estimating age [30, 31]. The result indicated the higher correlation with a non-linear relationship between pulp volume and age [30]. This finding suggested that pulp volume alone can be useful for age estimation.

The aims of this study were to investigate the relationship between chronological age and pulp volumes from a homogenous age distribution and to assess the effect of sex as predictor in age estimation.

## Materials and methods

### Sample selection

Ethical approval for the study was obtained from the Medical Ethical Committee of Advance Digital Imaging Centre Lahore Pakistan (16062017/2). All the CBCT images were recorded for the diagnostic and treatment purposes. Data was anonymized with only age, sex, and date of image recorded, and image-scanned details were provided. The exclusion criteria were as follows: Teeth with caries, wear, restoration, impaction, artifacts, periapical lesions, root resorption, teeth with open apex, evident wear and attrition, two roots and canals, and pulp calcification were excluded.

Sample size of scans was calculated with the help of the G*Power test software. Assuming a small effect size of *f*^2^ = 0.05, with an alpha level of .05, a target power of 95% and two predictors expected in the final model, the required sample size was found to be 776 scans.

**Fig. 1:**
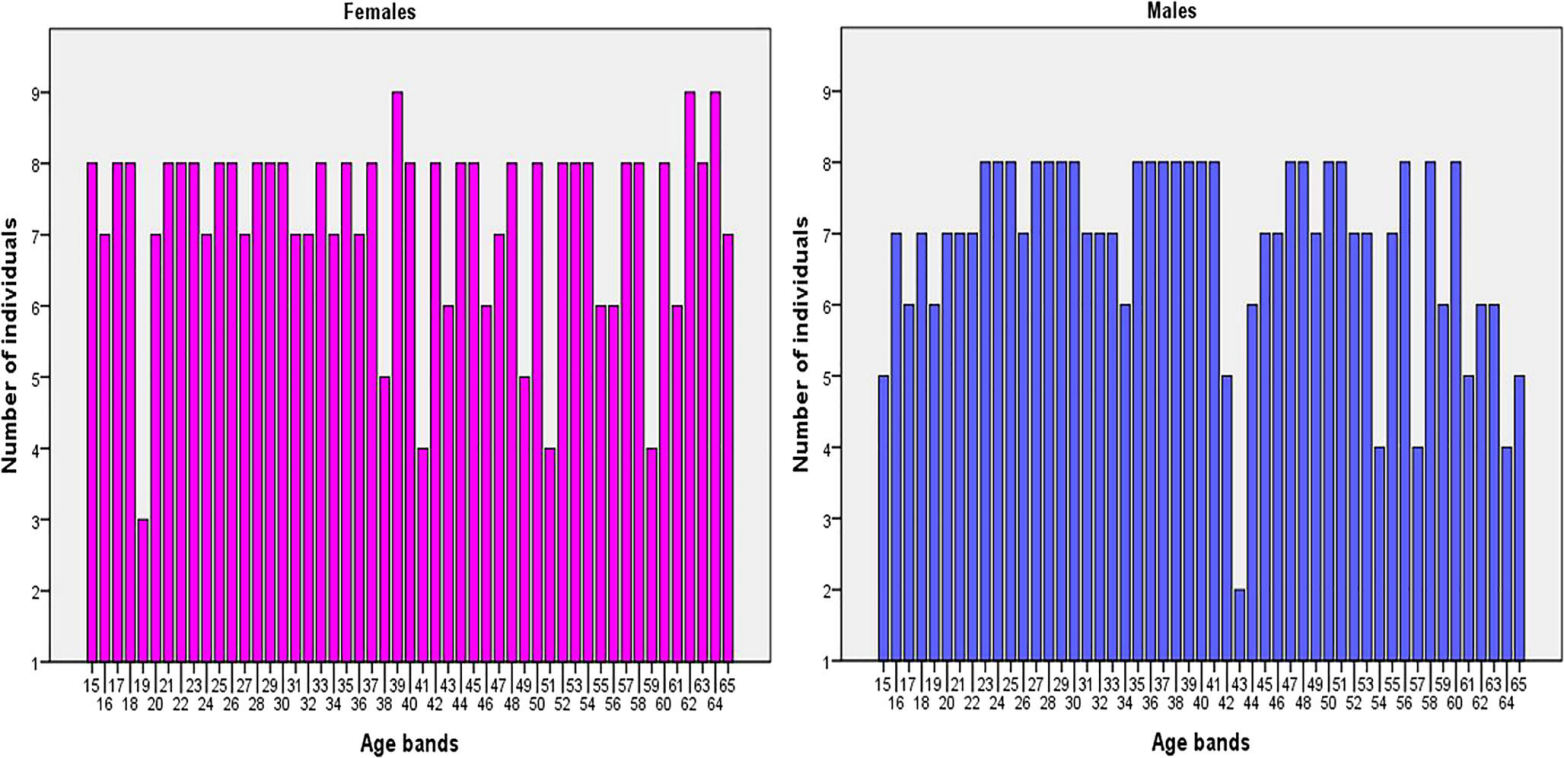
Distribution of sample size with age intervals and sex

**Fig. 2:**
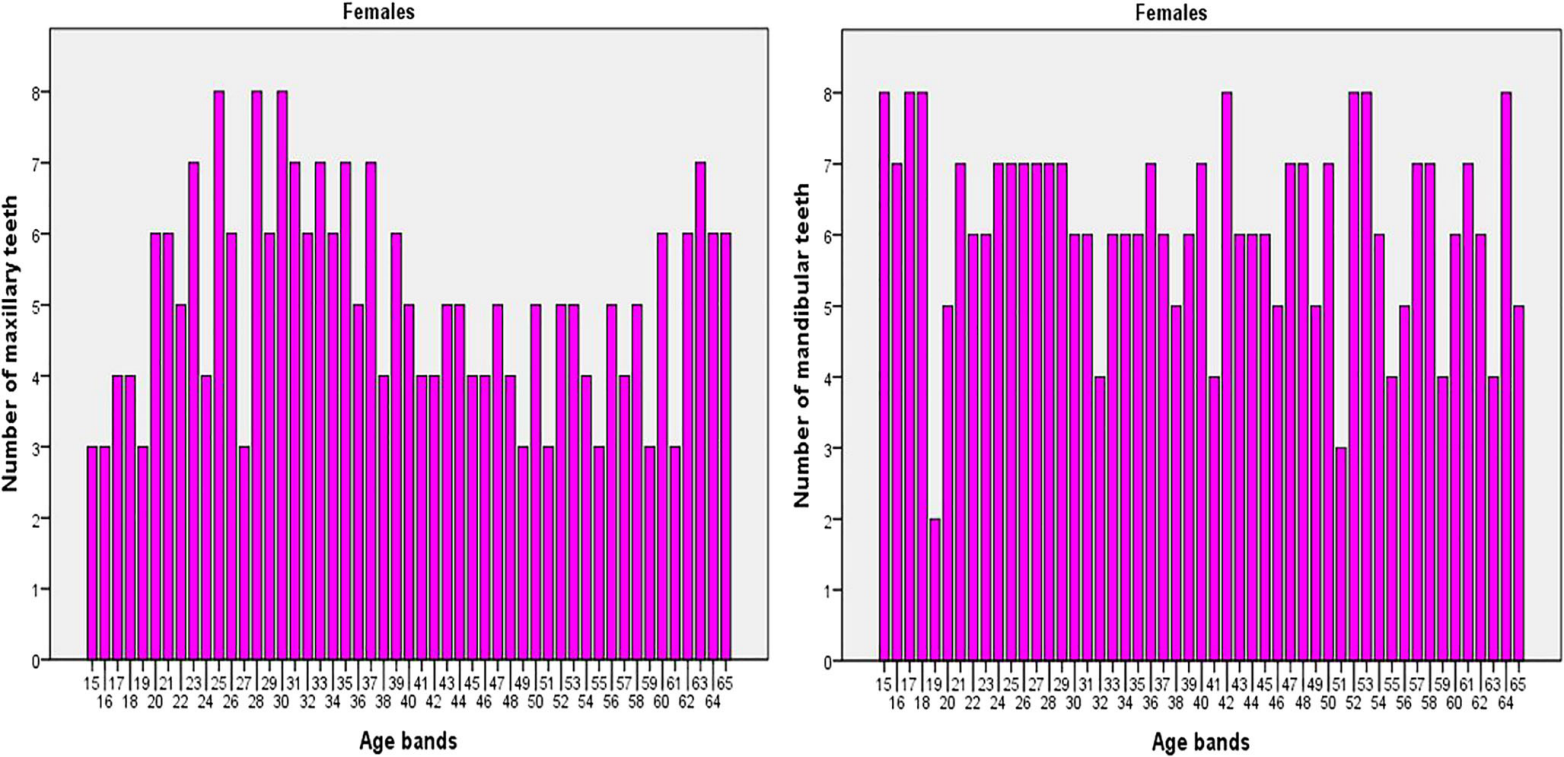
Distribution of maxillary and mandibular teeth by age in females

**Fig. 3:**
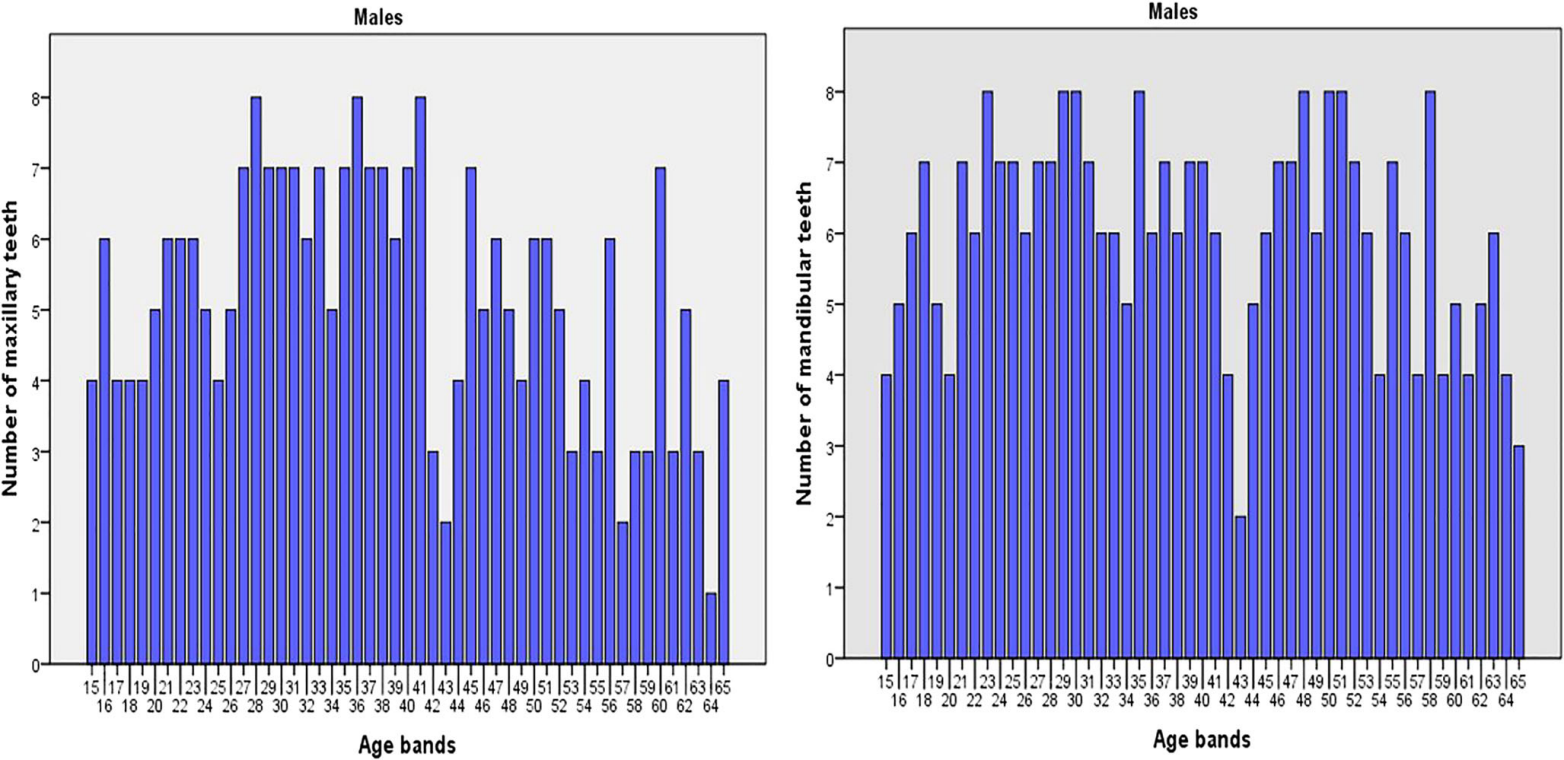
Distribution of maxillary and mandibular teeth by age in males

A total of 717 (349 males and 368 females) cone beam computed tomography (CBCT) images of left maxillary and mandibular canines were collected between December 2016 and September 2018 from the database of the Advance Digital Imaging Centre Lahore Pakistan. The sample was divided into 50 age groups with an interval of 1 year with maximum of 8 males and females in each age group (Fig. 1). The retrospective cross-sectional study consisted of 258 maxillary and 313 mandibular canines of females, and 263 maxillary canines and 307 mandibular canines of males aged 15–65 years (Figs. 2 and 3).

Based on the volumetric measurements, six predictive models (1–6) were proposed and sex was included in models 4, 5, and 6 (Table 1). To estimate the age, first, the relationship strength between age as a dependent and the different predictors as independent variables was calculated for the six models. The model with the highest *R*^2^ value was selected for the estimating age for the regression. The selected model was inspected for violations of the assumptions of multiple regression.

**Table 1:** Six models and predictors

Models	Predictors
Model 1	Left maxillary pulp volume
Model 2	Left mandibular pulp volume
Model 3	Left maxillary and mandibular volumes
Model 4	Left maxillary pulp volume and sex
Model 5	Left mandibular pulp volume and sex
Model 6	Left maxillary and mandibular volumes and sex

### Image acquisition

The reconstruction process of images mainly consists of two stages that are image acquisition and reconstruction stage. All images went through numerous steps in these two stages to form volumetric data. All the images were exported into Digital Imaging and Communication on Medicine (DICOM) format. The conversion (extension.dcm) was performed by the software Planmeca Romexis®.

All the CBCT images were acquired from the Planmeca ProMax 3D Classic CBCT unit (Planmeca, Helsinki, Finland) using 90-kVp tube current, 8-mA tube voltage,796.6 DAP dose area (mGy × cm2) and 12.038 scanning time. The voxel sizes of images were 200 μm and field of view selection was Ø8.0 × 8.0 cm (401 × 401 × 401) and focal spot was 0.5 mm. An open-source software (www.planmeca.com/softare/desktop/planmecca-romwxis) was used for calculating the volumes of pulp and tooth. The resulting data set imported into software Planmeca Rommexis®.

### Image interpretation

**Fig. 4:**
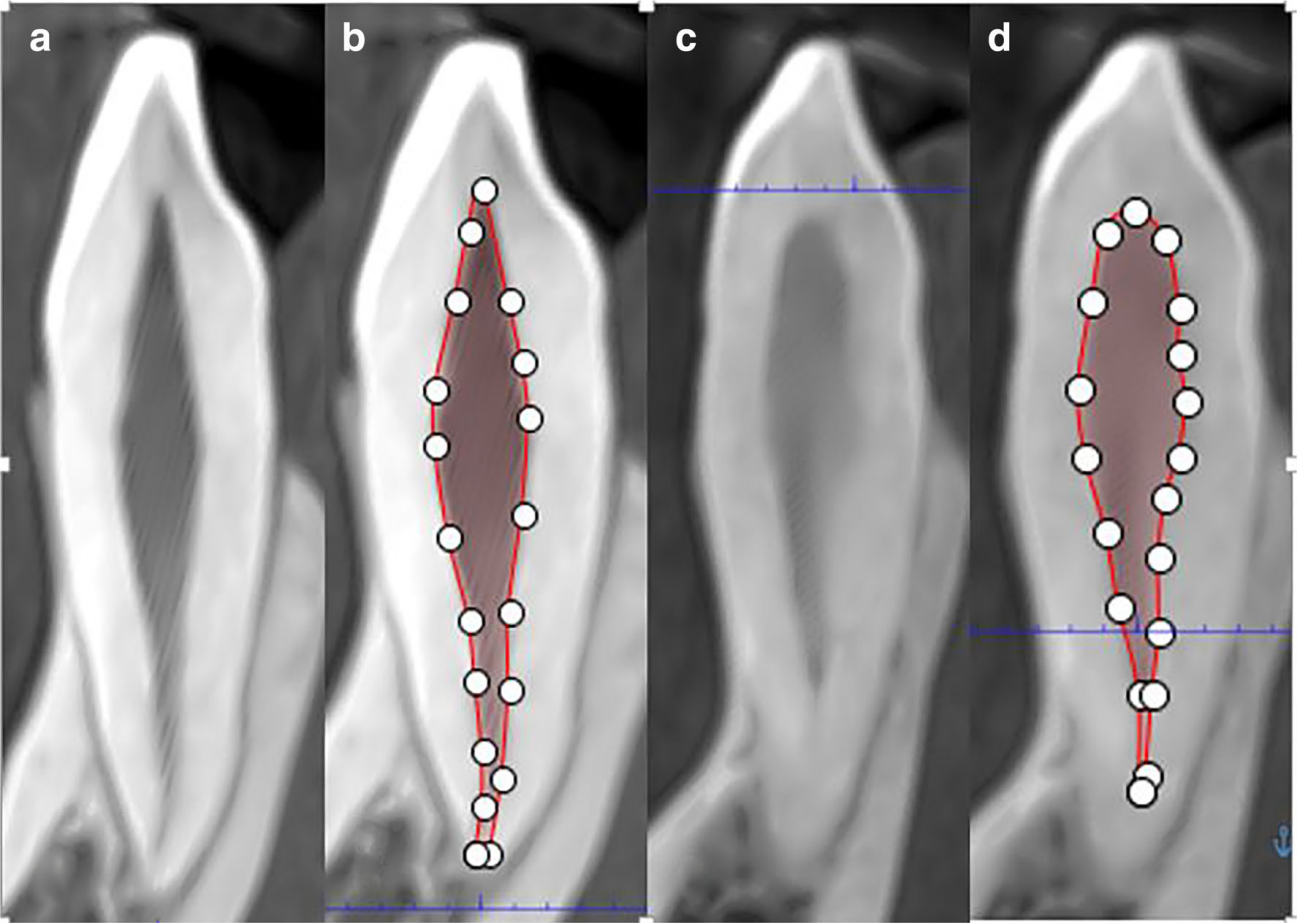
**a** Sagittal section of the upper left maxillary canine of 15 year old male. **c** 60-year-old male. **b**, **d** Traced outlines of the pulps

Each selected image was oriented into three (axial, coronal, and sagittal) planes in Planmeca Rommexis®. Manual segmentation was performed because of reliability and improvement in the apical region as compared with automatic segmentation [25]. After orientation of image in all three planes, the sagittal sections were selected for the pulp contour measurements. “Free region grow” icon was selected from annotation tools for the pulp volume measurements. A minimum of ten points were selected to define the pulp contour [1] (Fig. 4).

### Statistical analysis

Intra-class correlation coefficients (ICC 2, 1- consistency) were used to establish both test-retest and inter-rater reliability of the measurements. For test-retest reliability, 80 scans consisted of 105 canines were randomly selected with the time interval of the 3 weeks. For the inter-rater reliability, 30 scans consisted of 48 canines were randomly selected and calibrated by another expert examiner.

Analysis was performed using the R statistical programming language (version 3.2.2) and SPSS. The correlation between pulp volumes and chronological age was assessed using Pearson’s correlation coefficient. A *t* test was applied to compare the difference between pulp volumes of the males and females. A value of *p* < 0.05 was considered statistically significant for differences.

## Results

The results of the intra-rater and test-retest ICCs ranged between 0.945–0.9556 and 0.912–0.965 respectively. These results indicate an excellent consistency. The *R*^2^ for the different regression models is shown in Table 2.

**Table 2:** Results of the six models with *R*^2^ values

Models	Predictors	*R*^2^
Model 1	Left maxillary pulp volume	0.26
Model 2	Left mandibular pulp volume	0.26
Model 3	Left maxillary and mandibular volumes	0.22
Model 4	Left maxillary pulp volume and sex	0.31
Model 5	Left mandibular pulp volume and sex	0.33
Model 6	Left maxillary and mandibular volumes and sex	0.29

**Fig. 5:**
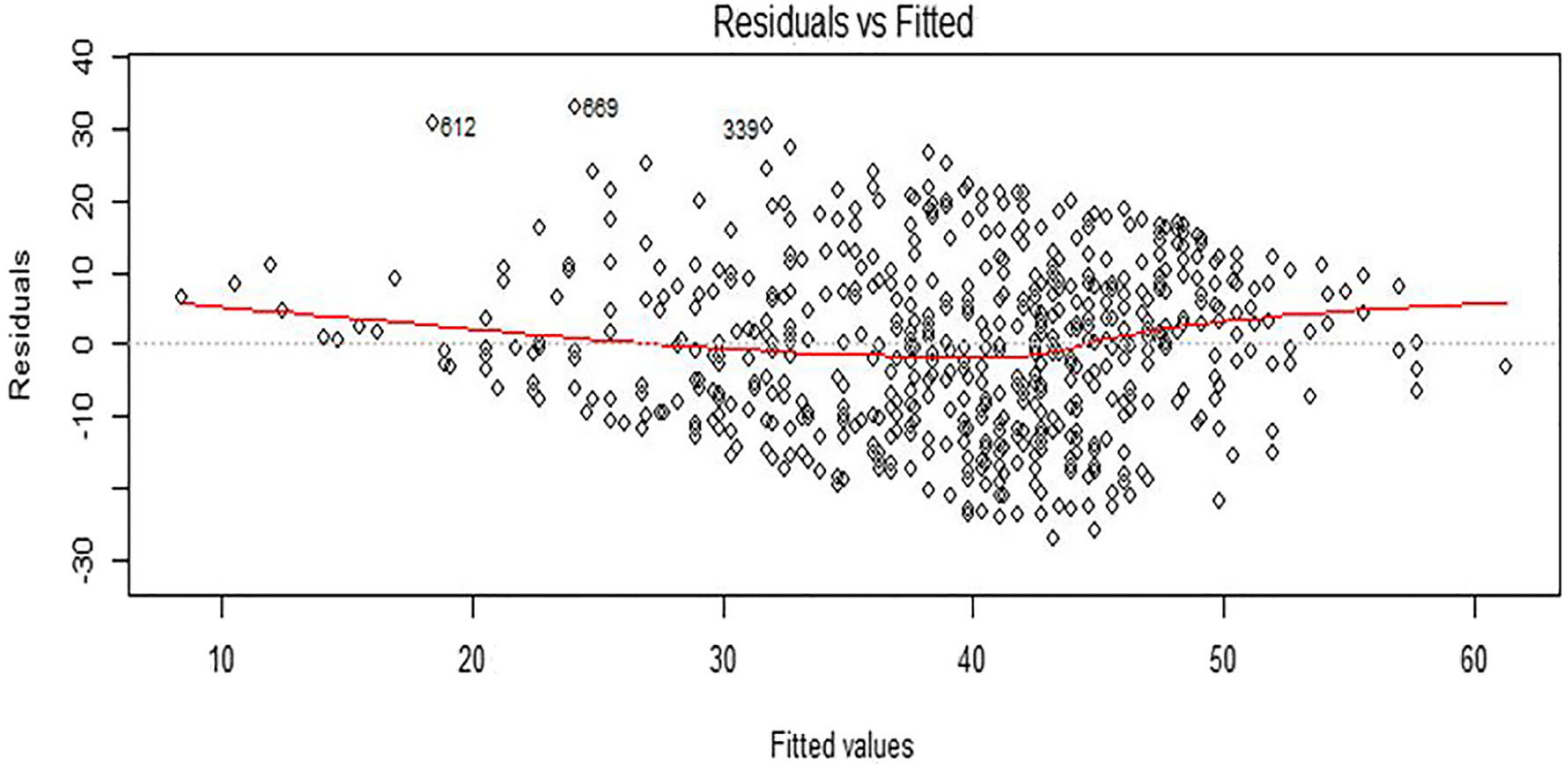
Plots of residuals against fitted values

**Fig. 6:**
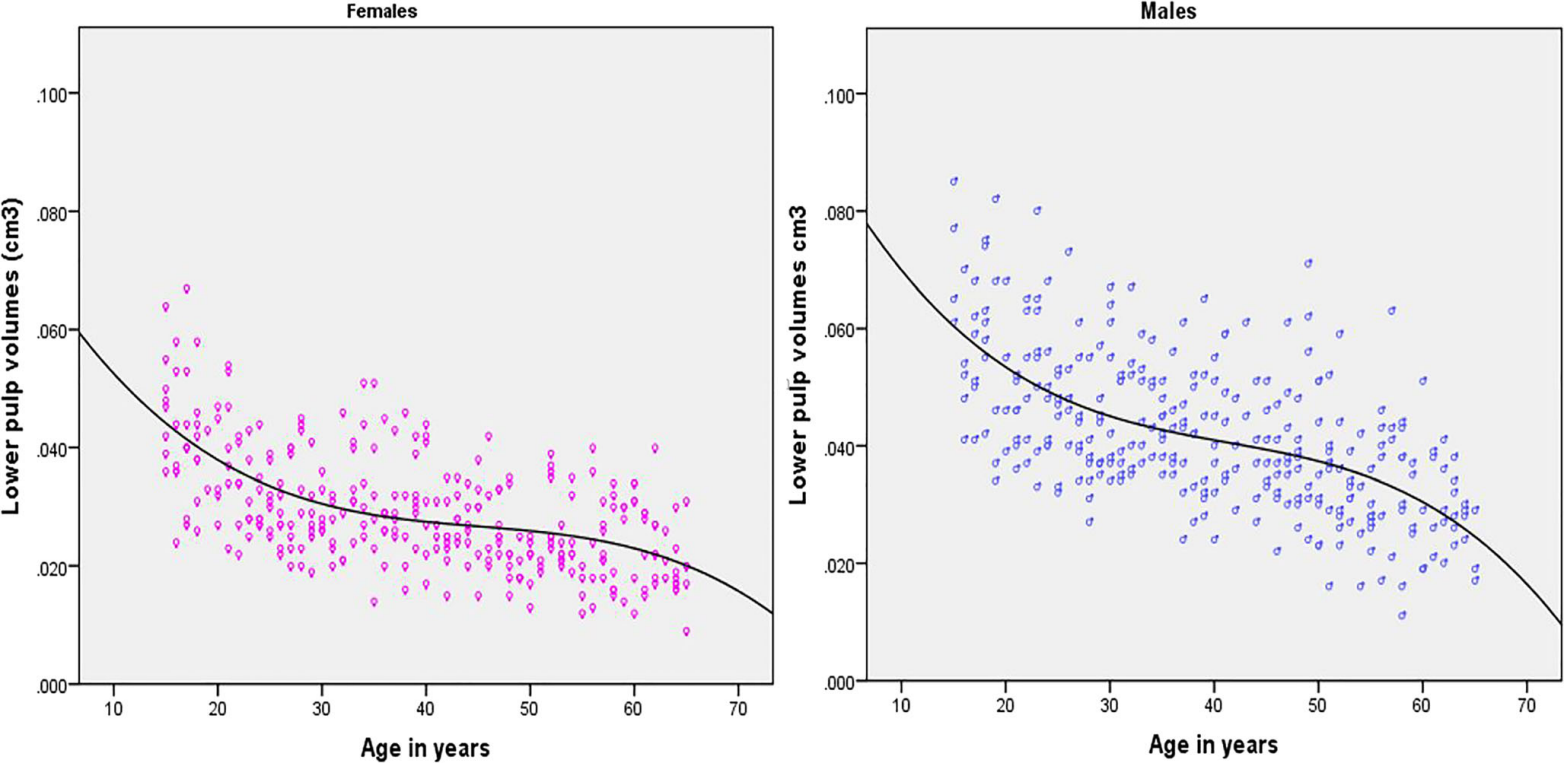
Scatter diagram showing the odd S-shaped non-linear relationship between model 5 and age

The results suggest that model 5 had the highest predictive power; therefore, model 5 was selected for analysis. The calculated volumes of model 5 ranged from 0.009 to 0.085 cm^3^ with a mean value of 0.0035 cm^3^ (SD = 0.01255) (Table 3). Model 5 was checked for extreme scores by calculating standardized residuals. No values exceeding ± 3 were detected so model 5 was considered free of outliers. Overly influential scores were sought using Cook’s distance. No values > 0.031 were found, indicating that no individual scores might bias the results. When checking the fitted*residuals* plot for model 5, there was a noticeable slope in both the upper and lower bounds of the data which was indicative of a problem with non-linearity (Fig. 5). This was confirmed with a Durbin Watson test which returned a significant *p* value indicating a problem with non-independence. The errors of model 5 are not random: when model 5 predicts someone is old, the errors are almost all in a negative direction indicating an over estimation of age and vice versa. This issue arises because the relationship between the predictors and age is not a simple linear one, but a rather more complex non-linear function (Fig. 6). While the prediction of model 5 remains rather better than just using the mean values to “guess” a person’s age, these problems call into question the appropriateness of using linear models to describe this relationship.

**Table 3:** Descriptive statistic of model 5 in pooled sex sample

Tooth	Sex	Maxillary teeth	Mandibular teeth	Minimum (cm^3^)	Maximum (cm^3^)	Mean	Std. deviation
Model 5	Males	263	307	0.0160	0.0820	0.04263	0.01255
	Females	258	313	0.0090	0.0640	0.02976	0.00924

The relationship between the pulp volume and age is shown in Fig. 6. An independent *t* test showed the difference in volumes of the pulp between males and females was statistically significant (*p* = 0.000). The dimorphism difference in the pulp volume remained obvious from the start of the juvenile age. The difference between males and females remains throughout the age; however, in old age the difference becomes narrowed.

The equation from the regression analysis was

Estimated Age = 60.370 + lower pulp volume × − 715.260 + sex × 8.791.

So, the equation adds 8.791 to a person’s estimated age when they are males.

The “oddity” in the distribution of the scores is only visible because of the large sample size. When experiments were conducted with repeating this process using a subset of our sample, the problems with non-linearity are obscured. This offers some explanation for why researchers with smaller sizes may have not found similar problems.

Given that applying a linear regression function to this clearly non-linear relationship is probably unwise, the descriptive statics for mandibular volume in each group are reported here (Table 4). Using these values, a basic calculator has been created in Excel which assesses whether a given score is consistent with membership of a given age group and sex. This calculator is included in the supplementary material.

**Table 4:** Descriptive statistics model 5 with age group and sex

Counts										
	15–19 group	20–24 group	25–29 group	30–34 group	35–39 group	40–44 group	45–49 group	50–54 group	55–59 group	60–65 group
Females	33	31	35	28	31	31	30	32	27	35
Male	27	32	35	32	34	24	34	33	29	27
Means										
	15–19 group	20–24 group	25–29 group	30–34 group	35–39 group	40–44 group	45–49 group	50–54 group	55–59 group	60–65 group
Females	0.043	0.035	0.030	0.031	0.030	0.028	0.025	0.024	0.023	0.023
Male	0.058	0.050	0.044	0.047	0.042	0.042	0.040	0.034	0.033	0.029
	Standard deviation									
	15–19 group	20–24 group	25–29 group	30–34 group	35–39 group	40–44 group	45–49 group	50–54 group	55–59 group	60–65 group
Females	0.011	0.009	0.007	0.008	0.009	0.007	0.007	0.006	0.008	0.007
Male	0.014	0.011	0.010	0.010	0.009	0.010	0.011	0.010	0.011	0.008

## Discussion

Despite having different contents and arrangements, the pulp and dentine have common embryonic origin. These two tissues share close relationship in terms of physiologic and pathologic reactions. Any one thing that disturbs the dentine will affect the pulp and vice versa. The odontoblasts are the most prominent cells of the pulp-dentine complex and are responsible for the dentine formation throughout the life [32, 33].

There are three types of dentine in the human tooth: primary, secondary, and tertiary dentine [33]. Primary dentine commences from odontogenesis until the tooth becomes functional and the formation of secondary dentine is produced immediately and continues throughout the life. The tertiary dentine is a reactionary dentine which is laid in specific regions in response to an injury [34]. It is a recognized fact that secondary dentine formation increases with age and as a result the volume of the pulp cavity shrinks. Therefore, the researchers calculated the pulp volumes and utilized it as predictor for estimating age [30, 31].

A non-linear relationship between pulp volumes and age was found in this study and the result is similar to those reported by Zhi et al. [30, 31]. The non-linear relationship appears to be the result of a variable rate change for pulp volume throughout life. It decreases more rapidly in early life, levels off in middle age, and resumes a more rapid descent in old age (Fig. 6). While the rate of change was somewhat variable, pulp volume declined consistently throughout the age ranges observed. This finding is consistent with the results which have observed a sharp decline volume in adolescent age [30, 31]. A novel finding however is that the relationship is not a simple linear one as reported previously by some papers, nor an easy to model 5 exponential relationship as reported by others, but rather an odd S-shaped function that is rather harder to model 5. The detection of this function by the authors and not others is entirely attributed to the large sample size used in present study.

There was a very high variability in pulp volumes within each age group in the present study. These findings were also observed in Zhi et al. studies [30, 31]. The heterogeneity of pulp volumes was observed in every age group in this study. A possible explanation for the differences in the pulp volume within a single age group in adolescent age is that it may be at least partially attributable to yet unanswered factors which affect primary dentine formation. It may be impossible to discriminate the primary dentine from secondary dentine on the CBCT images. To date, there have been no studies based on primary dentine and secondary dentine volumetric measurements. Further studies could be conducted to determine the possible effects on age estimation in adolescent age. Other possible explanations of variable pulp volumes in middle age groups are periodontitis, estrogen receptors present in pulp thus enhancing dentine formation, tooth shape and size, gene expression in odontoblasts, hormonal homeostasis, and occlusal stress [23, 35–37].One of the most notable differences observed in pulp volumes occur in those over 55 years of age, who demonstrate a rapid reduction in pulp volume this study contrary to other studies [30, 31].This might be attributed to the homogenous age distribution with large sample size. Again, discrepancies in the results of this study are most likely a product of the large sample size increasing the power of this study to detect small and subtle effects.

There are other possible explanations of the pulp volume inconsistencies. Venkatesh et al. findings suggested that pulp volumes are sensitive to orthodontics treatments [34]. In present study, scans of unknown history were utilized for estimating age. It is possible that variations in the pulp volumes might be due to orthodontics treatment especially in the early teenage years. Javed et al. evaluated the influence of the orthodontics treatment on human dental pulp and suggested that the link between orthodontic forces and dental pulp tissue has been insufficiently validated. However, the study did not focus on the pulp volumes as its primary outcome [38]. Future research could further clarify the effect of variable magnitude and duration orthodontic forces on the volume of the dental pulp tissue.

This present study results showed a significant difference in the pulp volumes of males and females. These results are similar to those reported by Zhi-pu et al. in 13 types of teeth except for mandibular first molars [31]. Similarly, other studies also reported a difference in volume between males and females [30]. The results of present study indicated higher *R*^2^ than the measured in single-root teeth [31]. However, their study reported a *R*^2^ value of 0.498, for maxillary second molar which was higher than present study [31]. Furthermore, another study reported a *R*^2^ value of 0.564 for maxillary first molars which was higher than present study [30]. This inconsistency in results was found because different methodologies and teeth were selected for pulp volume measurements.

The authors are not aware of any previous studies that assessed the role of sex as a predictor in age estimation in adults. Interestingly, the present study suggested that including sex as a predictor improves the predicative power of model 5. It is apparent from (Fig. 6) that sex-related difference was observed in the pulp volumes from the start of the adolescent age. Indeed, it appears it would be rather easier to estimate sex using pulp volumes than it was to estimate age. A possible explanation for the increased explanatory power might be that differences exist between males and females in primary dentine formation. Evidences show that the Y chromosome controls the thickness of dentine, whereas the X chromosome only controls the thickness of enamel [39].

In this study, it is possible to report that there was a rapid formation of secondary dentine observed in the mandibular canine until the 25–30 years age group. In middle age, secondary dentine deposition slows down and is consistent. In the 6th decade of age, rapid formation of dentine is once again observed. There was a significant difference between the pulp volumes of males and females and this difference remained throughout life. After 55 years of age, the difference narrowed. It might be assumed from this result that in old age the role of sex as a predictor of age becomes less informative.

Historically, there has been a broader focus placed upon the pulp-tooth ratio as a predictor for estimating age. These studies were mostly composed of images obtained from the μCT and cone beam computed tomography. Vandevort et al. were the first to report the moderate predictive relationship (*R*^2^ 0.31) between pulp-tooth ratio and age from 43 single-tooth μCT scans [21]. Similarly, Someda et al. measured the pulp-tooth ratios in five different regions of the 155 mandibular central incisors. The results of pulp-tooth ratio and pulp-tooth ratio excluding enamel showed almost the same correlation values with age. The higher *R*^2^ ranged from 0.66 to 0.78 [20]. Furthermore, Aboshi et al. calculated ratios of four different levels from 100 mandibular 1st and 2nd premolars. The coronal one-third of the root showed the significant correlation with age; however, the *R*^2^ from combined four levels showed the most significant correlation ranged from 0.635 to 0.703 [2]. In this study, the *R*^2^ of 0.33 was found, which was higher than that reported by Vandeevort et al. and lower than Someda et al. and Aboshi et al. [2, 21, 22].

The higher spatial resolution obtained with μCT increases the sensitivity of diagnostic tools derived from it. Despite this advantage, μCT results in a higher dose of radiation and more scanning time. In addition, extracted tooth required for the analysis [39].

Yang et al. were the first to correlate the pulp-tooth ratio with age from CBCT scans and obtained an *R*^2^ value of 0.29 from 28 different single-rooted teeth [24]. Similarly, the findings of Star et al. suggested an *R*^2^ value obtained from single teeth was low as compared with a combination of teeth [25]. The possibility of combing upper and lower pulp volumes was explored in the present study; however, this approach was rejected because it has a lower predictive power than individual tooth-pulp volumes. The combination of multiple tooth scores in a single model was also found to lead to problems with multicollinearity.

Pinchi et al. introduced a new method based on the geometrical shape of the pulp-tooth ratio of maxillary central incisors and obtained an *R*^2^ of 0.58 [26]. In addition, Adisen et al. used the pulp-tooth ratio of 131 maxillary canines to predict age and the result was *R*^2^ 0.486 [40]. Further analysis from Gulsahi et al. suggested that mandibular canine pulp-tooth ratio (*R*^2^ 0.210) was better than maxillary canine (*R*^2^ 0.153) [41]. The results from Biuki et al. ranged from *R*^2^ 0.65 to 0.75 for maxillary anterior teeth and *R*^2^ 0.60 to 0.76 for mandibular anterior teeth [18]. Marroquin et al. assessed the effect of sex on the regression models. His review concluded that majority of studies reported no effect of sex on age estimation. However, three studies reported that females had higher coefficient of determination than males [16], perhaps the most important reason for the higher coefficient of determination is that other studies used pulp + dentine volume/tooth volume ratio together as indicator for estimating age.

In the present study, a large sample with a balanced age distribution was used to investigate the relationship between pulp volumes and age. Information regarding systematic diseases, hormonal imbalance, or orthodontics treatment was not available. The authors suggest that a potentially fruitful future research strategy could include a history of the participants and determine the influence of other factors on pulp volumes. Moreover, the further studies on other tooth and methodologies are recommended for the improvement in the age estimation.

## Conclusions

The results indicated a strong relationship between maxillary and mandibular pulp volumes of canine. However, mandibular pulp volumes gave stronger relationship with chronological age as compared with maxillary pulp volume. The findings of this study indicated a non-linear relationship between mandibular pulp volumes and chronological age. The nature of the distribution suggests that this approach is most useful for aging very high or very low pulp volume scores. Scores in between the predicative power were relatively poor. The sampling technique and size highly affect the age estimation. The results indicated that including sex as a predictor improved the age estimation. In addition, there is a need to investigate the relationship between pulp volumes and age in other tooth.

## Electronic supplementary material

ESM 1 (XLSX 964 kb)
